# Structural Performance of EB-FRP-Strengthened RC T-Beams Subjected to Combined Torsion and Shear Using ANN

**DOI:** 10.3390/ma15144852

**Published:** 2022-07-12

**Authors:** Ahad Amini Pishro, Zhengrui Zhang, Mojdeh Amini Pishro, Wenfang Liu, Lili Zhang, Qihong Yang

**Affiliations:** 1 Civil Engineering Department, Sichuan University of Science and Engineering, Zigong 643000, China; ahad.ap@suse.edu.cn (A.A.P.); zhangzhengrui@suse.edu.cn (Z.Z.); wf_liu@suse.edu.cn (W.L.); zhanglili@suse.edu.cn (L.Z.); 2 School of Architecture and Design, Southwest Jiaotong University, Chengdu 610031, China; mojdehamini@my.swjtu.edu.cn; 3 School of Mathematics, Sichuan University, Chengdu 610065, China

**Keywords:** ANN, FRP, RC T-beams, combined loading, FEM, MSE

## Abstract

This research study applied Artificial Neural Networks (ANNs) to predict and evaluate the structural responses of externally bonded FRP (EB-FRP)-strengthened RC T-beams under combined torsion and shear. Previous studies proved that, compared to reinforced concrete (RC) rectangular beams, RC T-beams performance in shear is significantly higher in structural analysis and design. The structural response of RC beams experiences a critical change while torsion moments are applied in load conditions. Fiber Reinforced Polymer (FRP) is used to retrofit the structural elements due to changing structural design codes and loadings, especially in earthquake-prone countries. We applied Finite Element Method (FEM) software, ABAQUS, to provide a precise numerical database of a set of experimentally tested FRP-retrofitted RC T-beams in previous research works. ANN predicted structural analysis results and Mean Square Error (MSE) and Multiple Determination Coefficients $\;(R^{2})$ proved the accuracy of this study. The *MSE* values that were less than 0.0009 and $R^{2}$ values greater than 0.9960 showed that the ANN precisely fits the data. The consistency between analyzed experimental and numerical results demonstrated the accurate implication of ANN, MSE, and $R^{2}$ in predicting the structural responses of EB-FRP- strengthened RC T-beams.

## 1. Introduction

In recent decades, the external retrofitting of RC structures has become widespread in civil construction and academic studies [1,2,3,4,5]. In this application, composite materials are bonded to the surfaces of RC components to improve their structural capacity and performance, such as strength, stiffness, and ductility [6,7,8]. A review of the previous studies shows that many experimental investigations have been directed to study the behavior of RC beams retrofitted with externally bonded FRP (EB-FRP) composites such as carbon fiber-reinforced polymers (CFRP) with the primary schemes of U-wrapped and side-bonded plates or strips [1,2,7,8,9]. This is due to the advantages of FRP, such as its high specific strength, minor change in dead weight and geometry of the structures, and flexibility with different shapes.

External shear reinforcement of RC components can be achieved by three methods, namely side bond (SB), U-wrap (UW), and full wrap (FW). In SB, the FRP composites are attached only to the side faces of the RC beams, while in UW, the FRPs are connected to all three sides except the top face. On the other hand, at FW, the FRP is wrapped around the entire cross-section. Among these three typical strengthening schemes, the effectiveness of the FW scheme is the highest. It can be applied to RC rectangular RC beams or RC columns with access to all four sides. However, applying the FW configuration for monolithic cast plate beam structures such as RC T-beams is impossible because it is impractical for either drilling the slab or cutting longitudinal grooves in the web. To overcome these difficulties, lateral bonding or U-wrapping is generally used for slab–girder structures. However, the effectiveness of the UW configuration is higher than that of the SB scheme [10]. Typically, the experimental studies of the RC T-beams are slab-on-beam structures that are relatively more stable in shear than rectangular RC beams [11,12]. Bending moments and shear forces are considered primary in most structural designing, while torsion is deemed a secondary effect. However, torsion becomes a prominent effect when, for example, spandrel or curved beams are involved. Most specimens in the previous experimental research literature were EB-FRP-strengthened RC rectangular or box beams under monotonic torsion [13,14,15,16,17].

Many structural elements are under combined loading, such as torsion and bending, and torsion and shear. Some studies have studied RC T-beams retrofitted using various methods under pure torsion and combined torsion and shear [18,19]. The American Concrete Institute (ACI) [20] and Canadian Standards Association [21] cover most cases of retrofitting structures under combined bending and shear but do not provide enough points for strengthening reinforced concrete beams subjected to combined torsion and shear. Some proposed models [13,14,18] based on FIB [22] offered effective strain prediction, mostly involving shear than torsional tests [23]. However, it is necessary to build a comprehensive database of experimental studies on EB-FRP-strengthened RC T-beams subjected to combined torsion and shear. However, since experimental works are time and money-consuming, Finite Element Analysis (FEA) with commercial software is a valuable complement for investigating the structural behavior of retrofitted RC beams. Numerical studies on the effect of FRPs on structural behavior and the torque-torsional curves of RC beams have been carried out using FEA software such as ABAQUS, ANSYS, DIANA, Algor SAP, etc. [13,24,25].

L. Bernardo et al. [26] researched Generalized Softened Variable Angle Truss Model (GSVATM) to investigate the responses of RC beams under combined torsion and axial forces. Their theoretical prediction on $\left(M_{T}-\theta \right)$ followed their FEM models of reference beams. K. Protchenko et al. [27] studied the effect of high temperature on the structural capacities of RC members retrofitted internally with FRP reinforcements. The thermal expansion coefficient of carbon fibers leads to FRP bars′ creep, which causes the beams′ prestressing feature. In this case, they observed that the deflection started to decrease after a certain temperature threshold. R. Salih et al. [28] investigated the effect of FRP on the cyclic behavior of RC beams with openings. Their experimental results proved the significant effect of FRP sheets on increasing the structural capacities of their RC beam specimens. A parametric study by ABAQUS on the bond length and opening size was also considered in their study. M. Alembagheri et al. [29] conducted numerical research on strengthened and non-strengthened circular hollow section (CHS) T-joints subjected to axial forces. Their research studied the welding effects on joint buckling instability in compression and plastic failure in tension, which increased the plastic failure potential of the welding process. M. Chellapandian et al. [30] provided comprehensive research on the FRP retrofitting techniques for the rapid repair of pre-cyclic-damaged reinforced concrete (RC) and plain concrete (PC) beams. Their experimental and FEM studies proved that FRP retrofitted square columns have higher strength and stiffness under compression. D. A. Pohoryles et al. [31], in their experimental and parametric FEM analysis, proved the effect of FRP and textile reinforced mortars (TRM) in enhancing the structural capacities of retrofitted RC T-beams.

Recently, the application of Artificial Intelligence (AI) and neural networks experienced a remarkable progression in developing many engineering research studies. Maher G. M. Abdolrasol et al. [32] conducted a comprehensive study on the ANN application and some practical optimization techniques such as Particle Swarm Optimization (PSO), Genetic Algorithm (GA), Backtracking Search Algorithm (BSA), Artificial Bee Colony (ABC), Whale Optimization Algorithm (WOA), and Lightning Search Algorithm (LSA). Their research provided technical results for enhancing the ANN’s performance by applying the mentioned optimization techniques. Abdalrhman Milad et al. [33] presented three methods of ensemble Machine Learning (ML) for FRP strain prediction, including the effective parameters such as strength properties, material geometry, FRP properties, strain properties, and confinement properties. These parameters provided five input combinations to predict the strain enhancement ratio of FRP composites. Their research proposed Multivariate Adaptive Regression Spline (MARS), Extreme Gradient Boosting (XGBoost), and Random Forest (RF) as accurate alternative computer aid models for developing engineering strain-based problems. J. Amani and R. Moeini [34] applied Artificial Neural Network (ANN), Adaptive Neuro-Fuzzy Inference System (ANFIS), and Multi-Layer Perceptron (MLP) to predict the shear strength of RC beams. They compared their results with the Iranian Concrete Institute (ICI) and American Concrete Institute (ACI) codes. Their research proved the accuracy of ANN applications in structural behavior prediction.

In our previous studies [35,36,37,38,39], we applied the ANN and concepts of applied math such as Multiple Linear Regression (MLR) to investigate the Local Bond Stress (LBS) between steel bars and Ultra-High-Performance Concrete (UHPC). The research provided an accurate LBS equation and a precise comparison between experimental and numerical databases. It was proved that the application of ANN could deliver accurate results to the structural performance of RC members. In our recent study [40], we conducted a numerical investigation on the structural strength of FRP-Retrofitted RC Beams under Combined Torsion and Bending. The research considered the application of ANN to predict and verify the behavior of RC retrofitted beams. The outcomes proved an excellent consistency between the experimental FEM and ANN results.

This numerical research studied the structural behavior of RC beams strengthened by externally bonded carbon fiber reinforced polymers (CFRPs) subjected to combined torsion and shear. To provide an accurate database, we applied FEM software ABAQUS CAE (Version 6.10.1; Karlsson & Sorensen Hibbitt Inc.: Pawtucket, RI, USA) [41] to calibrate the experimental results of six scaled RC T-beams (two reference beams and four EB-FRP-strengthened beams) that existed in previous research [19]. Then, our database was used to train the Artificial Neural Network (ANN) to predict and evaluate the structural responses and effectiveness of various EB-FRP schemes and significantly extended the U-jacket of our numerical specimens. Finally, Mean Square Error (MSE) and Multiple Determination Coefficients $\left(R^{2}\right)$ verified the accuracy of this research. Since there is no accurate analytical equation to determine the behavior of EB-FRP-strengthened RC T-beams under combined torsion and shear, this research applied the ANN to predict and evaluate the structural responses of this type of RC beam.

## 2. Material Modeling and Nonlinear Analysis

Six T-shaped RC beams, TB1, TB1S1, TB1S2, TB1S3, TB3, and TB3S4, were experimentally investigated under combined torsion and shear [19]. Instruments monitored the applied load and the resultant deformation of the specimens. The employed wrapping configurations of CFRP strips are depicted in Table 1. The CFRP sheets, bidirectional fabrics, were applied within the T-beam test region. The fibers were oriented in the ±45° direction to the T-beams longitudinal direction to provide the maximum strength in torsion and shear.

The measured mean concrete strength, the particular aspects, and a summary of these specimens are listed in Table 2.

The experimentally and analytically determined properties of CFRP are reported in Table 3.

In this study, we defined the response of materials based on the previous experimental results. Due to the FRP fracture of specimens’ failure, the Hashin damage model was applied in ABAQUS [42] to model FRP’s damage and failure explicitly. To define the damage evolution, we selected the fracture energy of 0.01. For the steel bars, isotropic elastic strengthening was considered. Equations (1) and (2) present the actual stress and plastic strain to define the response of materials.

(1) $$\sigma_{true}=\sigma_{nom}\left(1+\varepsilon_{nom}\right)\;$$

(2) $$\varepsilon_{ln}^{pl}=\ln\left(1+\varepsilon_{nom}\right)-\frac{\sigma_{true}}{E}$$

To model the concrete response in the ABAQUS, we applied the damage plasticity model [43]. Poisson’s ratio, tensile strength $f_{ct}$, and the modulus of elasticity E were determined based on the previous technical reports [44,45,46,47,48,49].

The linear eight-node brick elements discretized the concrete beam. Moreover, linear truss elements discretized the longitudinal reinforcing bars and the steel stirrups embedded in the concrete. The spacing between the longitudinal reinforcing bars is 20 mm. To simulate the actual response for RC beams, we considered no relative displacement between steel reinforcing bars and concrete (embedded option in ABAQUS). This technique specifies that a group of elements is embedded in a host element. ABAQUS finds the geometric relationships between the nodes of the host and the embedded elements. CFRP strips were modeled by choosing the linear four-node elements and tied to the corresponding 3D eight-node cohesive elements. The interface between the concrete and FRP was assumed as a cohesive zone. Because of the nonmonotonic stress–strain responses of materials [50], we applied an explicit dynamics solver in ABAQUS to conduct the nonlinear analysis. In addition, we set the slow displacement rate and the kinetic energy as not more than 2% of the internal energy.

**Figure 1:**
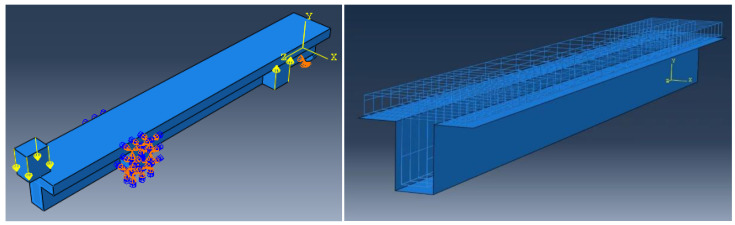
T-shaped beam, CFRP, and steel bars modeling in ABAQUS.

The mean measured specimen geometries were utilized to simulate the test specimens. The entire 3D geometry of the concrete volume and FRP strips was modeled with 3-D brick-type F.E. elements. The FRP interface and the concrete were simulated as a cohesive zone with a suitable traction separation response. Using the Tie option of ABAQUS allows a model of two different surfaces (FRP and Concrete) to have two separate motions from each other. In other words, two separate motions for master and slave surfaces at their interface have been considered [40]. Figure 1 shows the modeling of a T-beam and the CFRP and steel bars positioning for TB1S2 in the ABAQUS.

## 3. Results and Discussions

### 3.1. Evaluation of Strengthened RC T-Beams under Combined Torsion and Shear

According to the numerical analysis results of beams, cracking happened earlier in the reference beam (TB1) compared to the other beams, and the ultimate torque of TB1S3 is higher than the others. Table 4 shows the experimental and numerical results of cracking torque, ultimate torque, and maximum angle of twist for the beams TB1, TB1S1, TB1S2, TB1S3, TB3, and TB3S4. Comparing these experimental and numerical values indicates that using CFRP strips increases the torsional capacity of RC beams.

The comparison of numerical and experimental values of cracking torque, ultimate torque, and ultimate angle of twist of T-beams under combined torsion and shear is presented in Table 5. As can be seen in similar data, there is acceptable conformity in experimental and numerical results. Moreover, the critical shear, ultimate shear, and comparison of these results for beams TB1, TB1S1, TB1S2, TB1S3, TB3, and TB3S4, are illustrated in Table 6. From the outcome point of view, there is desirable coordination between experimental and numerical values. Therefore, it can be mentioned as further proof of the reliability of numerical analysis and FEM software (ABAQUS) to foretell the behavior of these kinds of studies. The last two columns of this table illustrate the acceptable approach to numerical and experimental results.

### 3.2. Application of ANN

**Figure 2:**
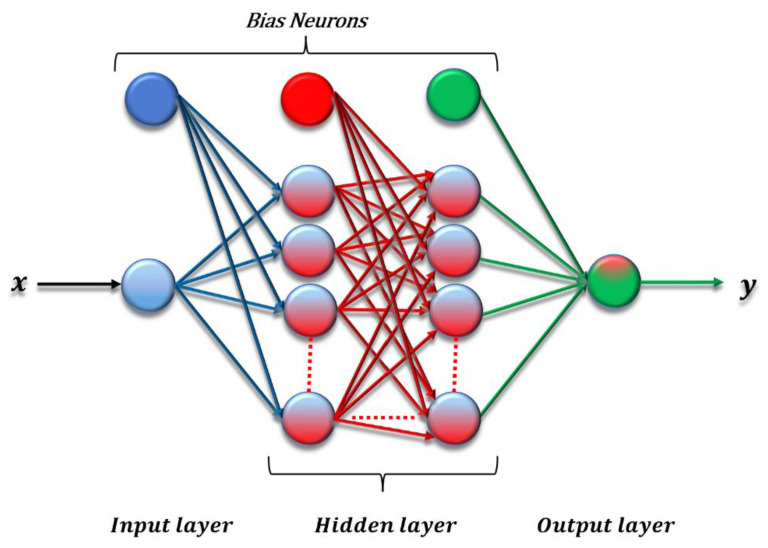
ANN structure.

ANN provides functional approaches to predict the results and scientific approximations, classify problems, and check the accuracy of research outcomes. As Figure 2 presents, three layers construct the main body of the ANN.

In our experiments, one input neuron x denotes torque or shear force, and one output neuron y denotes strain or angle of twist. For the sake of simplicity, we do not show the activation function in the figure and use the same one between two adjacent layers. The system of ANN connects the input to output in one-way information processing. The error value is calculated after receiving the input data by ANN. There are groups of neurons in each layer that have their importance by assigned weight. The backpropagation algorithm leads to learning and solving errors based on the data of input and output layers. Mean Square Error (MSE) provides a valuable loss function for regression problems to predetermine the logical minimum error [39,40].

(3) $$MSE\left(y,\;y^{\prime }\right)=\frac{\sum_{i=1}^{n}{\left(y_{i}-y_{i}^{\prime }\right)}^{2}}{n}\;$$

**Figure 3:**
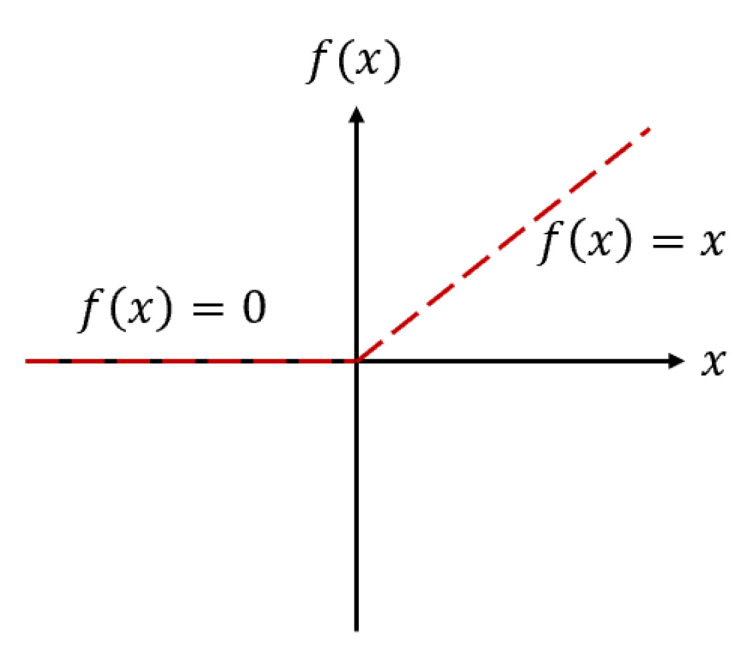
The neurons′ activation function (f).

Figure 3 presents the neurons′ activation function (f) to support the rectified linear unit (ReLU) function applied in this study.

(4) $$ReLU\left(x\right)=\{\begin{array}{c} x\;if\;x>0\\ 0\;if\;x\leq 0 \end{array}\;$$

**Figure 4:**
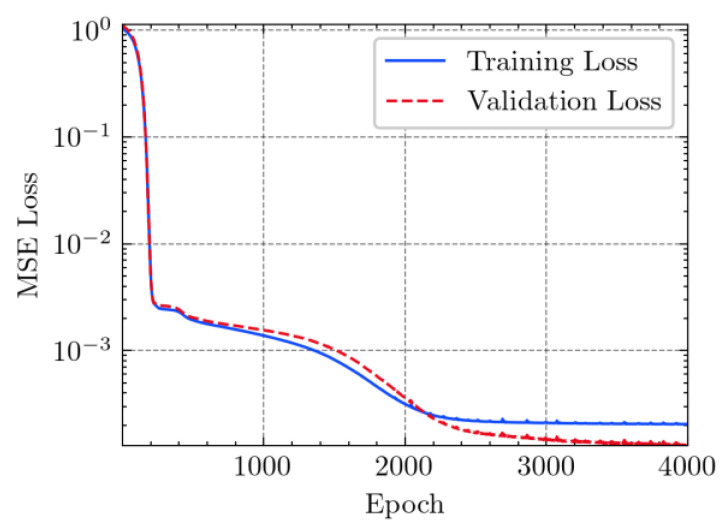
Loss function, MSE.

Equation (5) presents the output signal $\left(y_{k}^{l}\right)$ of neuron k in the l-th layer. In this equation, f denotes the activation function, $u_{k}^{l}$ represents the linear output in the l-th layer, and $b_{k}^{l}$ indicates the bias in the l-th layer. Equation (6) calculates the linear output in which $w_{ki}^{l}$ and $x_{i}^{l}$ stand for interconnection weights and input signals in the l-th layer, respectively [39,40]:

(5) $$y_{k}^{l}=f\left(u_{k}^{l}+b_{k}^{l}\right)$$

(6) $$u_{k}^{l}=\overset{N}{\underset{i=1}{\sum }}w_{ki}^{l}\;x_{i}^{l}$$

where N means the number of neurons in the (l−1)−th layer. Regarding scientific applications of deep learning methods [51,52,53,54,55,56,57], we applied the *MSE* loss function, as shown in Figure 4. Noting that, we randomly split the data into training datasets (80%), validation datasets (10%), and testing datasets (10%). Adaptive moment estimation (ADAM), a practical algorithm in deep learning, was used to optimize the convergence and improve the model. In the ANN, we used four hidden layers, and each hidden layer had eight neurons. Noting that, for each neuron, different biases were applied. The Xavier initialization technique initialized the weights and biases.

### 3.3. Model Evaluation Method

To verify the regression equations’ accuracy of fit in the multilinear regression method, we applied the Mean Square Error (MSE), as shown in Equation (7).

(7) $$MSE=\frac{1}{n}\overset{n}{\underset{i=1}{\sum }}{\left(y_{i}-\hat{f}\left(x_{i}\right)\right)}^{2}$$

The Multiple Determination Coefficients ($R^{2}$) is another indicator to evaluate the convergence of multilinear regression equations. $R^{2}$ reflects the proportions described by the estimated regression equations in the variance of the factor variable y, calculated as the proportion of progression squares to the sum of total squares. In Equations (8)–(10), SST represents the total sum of squares, SSR denotes the regression sum of squares, SSE indicates the error sum of squares, ${\hat{y}}_{i}$ stands for the model forecast value, and $\bar{y}$ shows the average of y [39,40].

(8) $$SSR=\overset{m}{\underset{i=1}{\sum }}{\left({\hat{y}}_{i}-\bar{y}\right)}^{2}$$

(9) $$SSE=\overset{m}{\underset{i=1}{\sum }}{\left(y_{i}-\bar{y}\right)}^{2}$$

(10) $$R^{2}-\frac{SSR}{SST}=1-\frac{SSE}{SST}\;$$

### 3.4. Comparison of ABAQUS and ANN Results

Based on the numerical analysis of our models, it can be confirmed that using externally bonded CFRP (EB-CFRP) strips has desirable effects and performances in increasing the ultimate strength and creating a significant decrease in the crack width and growth for beams TB1, TB1S1, TB1S2, TB1S3, TB3, and TB3S4.

Table 7 illustrates the structural responses and the ANN results of retrofitted T-beams under torsion and shear. In this Table, Figures (a) presents the FE models of the T-beams in ABAQUS. As observed from Figures (a), the cracking rise and growth of the FRP-retrofitted areas of these beams occur slowly as opposed to un-retrofitted areas. Using FRP sheets considerably affects cracking growth reductions in the beam subjected to combined torsion and shear, while the beam is twisted under loading. Significantly, the structural behavior of these beams can be precisely predicted using this research method, as shown in Figures (b) to (g). Figures (b), (d), (e), and (g) show that the numerical results are close to experimental results. Figures (b) and (e) show that the curve of numerical results can almost wholly overlap the curve of experimental results. In Figures (d) and (g), nearly all the data are close to the diagonal line, which means that the prediction results are immediate to the experiment results. Figures (c) and (f) present the low training loss and validation loss, and we can know that our models are not over-fitted.

The test region is the part along the beam between two load points. The strain was measured as the transverse steel’s load increased in this region. Figures (e) in Table 7 show the shear force–strain graphs of numerical analysis and experimental study for reference and retrofitted beams. As the graphs show, while the load is applied on these beams, the transverse steel experiences an increase in length called strain. When cracking occurred, the concrete carried a lesser percentage of load while the transverse steel tolerated a greater load, which was more desirable than cracked concrete. The structural behavior of transverse steel in load tolerance has a limitation. Moreover, diverse behavior is seen because of different FRP configurations and orientations. Figures (e) shows a significant overlap of numerical and experimental results.

The CFRP full wrapping retrofitting technique is relatively more effective than the extended CFRP U-jacket. Although applying the CFRP U-jacket retrofitting technique is quite convenient for most practical situations, its efficiency is the lowest among the other schemes. The extended CFRP U-jacket increased the structural performance of the beam to sustain more twists compared to the CFRP U-jacket. This enhancement is because of the CFRP extension to the flange, which decreases the flange crack growth. The CFRP full wrapping is the most effective retrofitting technique. This retrofitting technique provides complete wrapping around the sections, which would be more applicable for the new beams. In contrast, accessing all sides of the existing beams is difficult. Therefore, successfully applying the flange jacket for the CFRP full wrapping technique in practical cases is not easy.

Training Set *MSE* is calculated from the training data, while the test error (square error) data provides the Test Set *MSE* [39,40]. According to these data, *MSE* is very small if the predicted value response is close to the real value response. The proper model should be selected to minimize the test square. Table 8 provides the statistical parameters of our ANN model.

As shown in Table 8, the ANN perfectly fits the data. The *MSE* values of beams TB1, TB1S1, TB1S2, TB1S3, TB3, and TB3S4 subjected to torsion in the training set are 0.000218, 0.000010, 0.000855, 0.000120, 0.000012, and 0.000015, respectively. It can be observed that all of these results are reasonably accurate and less than 0.000900. Furthermore, the *MSE* in the test set also presents the values of 0.000216, 0.000010, 0.000854, 0.000119, 0.000011, and 0.000016 for the mentioned beams, respectively. Therefore, the results in the test set are also less than 0.000900. Moreover, the *MSE* data of the beams subjected to shear are precisely less than 0.000900 and acceptable.

Considering the $R^{2}$ values in Table 8, regardless of whether the retrofitted T-beams are subjected to torsion or shear, all the training set results are close to 1. In addition, the test set $R^{2}$ values of TB1, TB1S1, TB1S2, TB1S3, TB3, and TB3S4 under torsion and shear present 0.999572, 0.999934, 0.998567, 0.999617, 0.999861, 0.999996, 0.999742, 0.998732, 0.996277, 0.999500, 0.999677, and 0.998963, respectively, which means that the ANN precisely fits the data.

Regarding the results presented in Table 7 and Table 8, it can be concluded that applying the machine learning concept, particularly ANN, would provide a precise prediction of structural behavior for EB-FRP-strengthened RC beams. Moreover, $R^{2}$ and MSE, the metrics of applied mathematics, would be the logical tools used for checking the accuracy of structural analysis.

For the developed ANN model of this study, we used the initialization process to set the initial values of weight. Applying an appropriate initializer would accelerate the convergence time and creates a lower loss function to enhance the model′s accuracy. Xavier Initialization, Zero Initialization, and Initialization with the same Random value are the principal techniques of weight Initialization [58,59]. In this research, we applied the Xavier Initialization to efficiently utilize the Sigmoid activation function.

The developed ANN model of this research consists of four hidden layers, each including eight neurons, noting that different biases were applied for each neuron. Table 9 presents the weights and biases operated by the Xavier initialization technique for the TB1beam.

Regarding Equations (5) and (6), the indicators denote the following:

$w^{l}$: the weights between the (l−1)-th and the l-th layers;
$b^{l}$: the biases between the (l−1)-th and the l-th layers;
$w_{ki}^{l}$: the weight from the k-th neuron in the (l−1)-th layer to the i-th neuron in the l-th layer;
$b_{k}^{l}$: the bias of the k-th neuron in the l-th layer.

In Table 9, $w^{l}$ and $b^{l}$ represent the values of a matrix and a vector, respectively.

## 4. Conclusions

This study applied the ANN in structural analysis. It used ABAQUS and Finite element analysis to investigate the increase in strength of retrofitted and non-retrofitted RC T-beams by different FRP orientations. The dimensions, end supports, and specifications of beam models were identical to those of the experimental studies. The FEM results provided a comprehensive database for the ANN to predict the structural responses of EB-FRP-strengthened RC T-beams subjected to combined torsion and shear. A comprehensive evaluation of the results extracted from the FEM analysis, the developed ANN model, MSE, and $R^{2}$ yielded the following significant developments:

1. Regarding the figures and tables, numerical cracking torques, ultimate torques, the maximum angle of twist, cracking shear, and ultimate shear values are consistent with the experimental results for all beams.
2. According to the numerical results, the ultimate structural strength of EB-FRP-strengthened RC T-beams depends on the volumetric ratio and fiber orientation of the employed FRP materials. For a certain angle of twist, beams with higher torsional reinforcement have greater torsional capacity, increased post-cracking stiffness, and ultimate angle of twist.
3. The applications of ANN, *MSE*, and $R^{2}$ can evaluate the consistency of experimental and FEM results.
4. The resultant values of *MSE* less than 0.0009 and $R^{2}$ greater than 0.9960 proved that the developed ANN model of this study fits the data precisely.
5. The research methodology presented in this study can be adopted and applied in expensive and time-consuming structural experiments subjected to combined torsion, shear, and bending.

## 5. Possible Directions for Future Studies

This research investigated the application of ANN, *MSE*, and $R^{2}$ to predict and evaluate the structural performance of EB-FRP-strengthened RC T-beams subjected to combined torsion and shear. However, providing a set of comprehensive equations using Multiple Linear Regression (MLR) to calculate the structural capacities of EB-FRP-strengthened RC beams under combined torsion, shear, and bending is still an open discussion for future studies. Moreover, applying algorithms such as GP, SVM, RF, etc., to conduct precise classifications and assessments of the results is essential.

## Figures and Tables

**Table 1:** Strengthening wrapping configuration.

Beam	Wrapping Configurations	Strengthening Schemes	
TB1	None (Un-retrofitted beam)	---	
TB1S1	U-Jacket	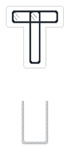	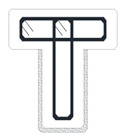
TB1S2	Extended U-Jacket	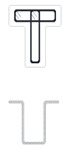	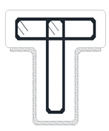
TB1S3	Full Wrapping	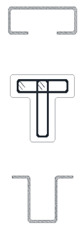	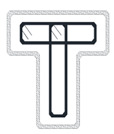
TB3	None (Control beam)	---	
TB3S4	Extended U-Jacket + Full Wrapping	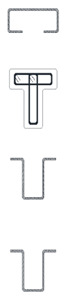	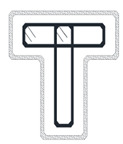

**Table 2:** Beam tests summary.

Designation	Combined Effects	Mean Concrete Strength (MPa)	FRP	Torsional Moment at First Crack (kN.m)	Maximum Torsional Resistance (kN.m)	Maximum Shear Resistance (kN)
TB1	Torsion & Shear	25	--	10.5	23	44
TB1S1	Torsion & Shear	25	CFRP	19.5	34	60
TB1S2	Torsion & Shear	25	CFRP	14	38	80
TB1S3	Torsion & Shear	25	CFRP	18	39	83
TB3	Torsion & Shear	25	--	3	10.75	109
TB3S4	Torsion & Shear	25	CFRP	16	17.5	173

**Table 3:** Material properties of CFRP.

FRP	E $\left(N/mm^{2}\right)$	Tensile Strength $\left(N/mm^{2}\right)$	Thickness (mm)
CFRP	63,300	609	0.86

**Table 4:** Cracking torque, ultimate torque, and maximum angle of twist for the beams TB1, TB1S1, TB1S2, TB1S3, TB3, and TB3S4 (experimental and numerical study).

Beam	$\;T_{crE}\;\;\left(kN.m\right)$	$\;T_{uE}\;\;\left(kN.m\right)$	$\;\theta_{uE}\;\;\left(Deg/m\right)$	$\;T_{crN}\;\;\left(kN.m\right)$	$\;T_{uN}\;\;\left(kN.m\right)$	$\;\theta_{uN}\;\;\left(Deg/m\right)$
TB1	10.5	23	2.6	10	23.38	2.31
TB1S1	19.5	34	1.5	17.5	33.85	1.33
TB1S2	14	38	2.5	16	39	2
TB1S3	18	39	2.5	15.5	40	2
TB3	3	10.75	0.9	3.3	10.44	1.01
TB3S4	16	17.5	8	14.5	18	8.5

**Table 5:** Comparison of numerical and experimental values of cracking torque, ultimate torque and ultimate angle of twist T-shaped beams subjected to combined torsion and shear.

Beam	$\frac{T_{crN}\;}{T_{crE}}$	$\frac{T_{uN}}{T_{uE}}$	$\frac{\theta_{uN}}{\theta_{uE}}$
TB1	0.95	1.016	0.888
TB1S1	0.897	0.995	0.886
TB1S2	1.14	1.026	0.8
TB1S3	0.861	1.025	0.8
TB3	1.1	0.971	1.12
TB3S4	0.906	1.028	1.062

**Table 6:** Cracking shear, ultimate shear, and comparison of these results for beams TB1, TB1S1, TB1S2, TB1S3, TB3, and TB3S4.

Beam	$V_{crE}\;\;\left(kN\right)$	$V_{uE}\;\;\left(kN\right)$	$V_{crN}\;\;\left(kN\right)$	$V_{uN}\;\;\left(kN\right)$	$\frac{V_{crN}}{V_{crE}}$	$\frac{V_{uN}}{V_{uE}}$
TB1	17	44	16.3	42	0.959	0.95
TB1S1	24	60	28	68.7	1.16	1.14
TB1S2	35	80	33	73.03	0.94	0.91
TB1S3	45	83	38	88.4	0.84	1.06
TB3	58	109	60	119.2	1.034	1.09
TB3S4	70	173	81	184.47	1.157	1.066

**Table 7:** Structural response and ANN results of T-Beams subjected to combined torsion and shear.

**Beam**	TB1	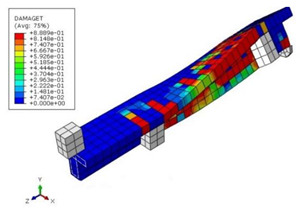 (a)		
**Structural Response**	Torsion	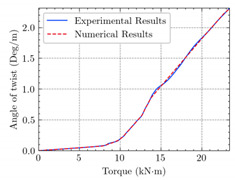 (b)	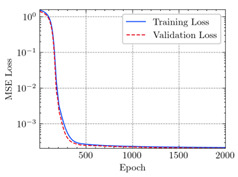 (c)	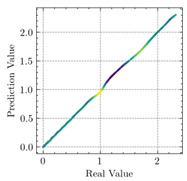 (d)
	Shear	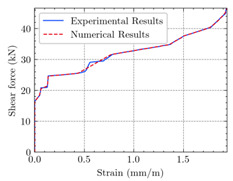 (e)	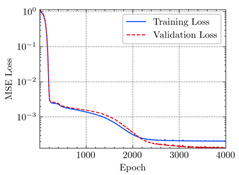 (f)	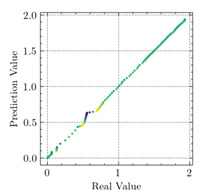 (g)
**Beam**	TB1S1	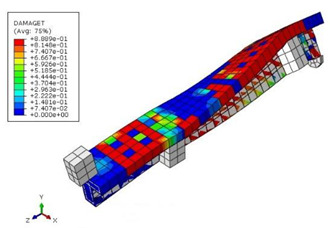 (a)		
**Structural Response**	Torsion	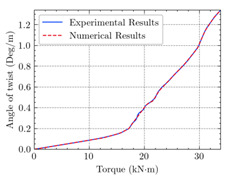 (b)	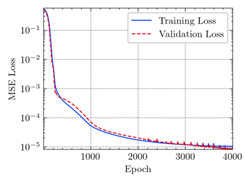 (c)	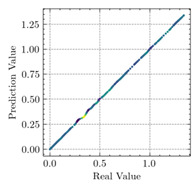 (d)
	Shear	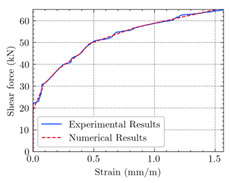 (e)	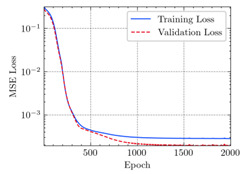 (f)	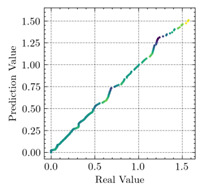 (g)
**Beam**	TB1S2	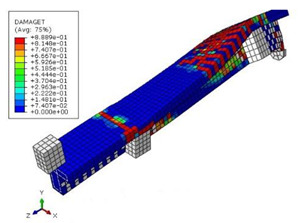 (a)		
**Structural Response**	Torsion	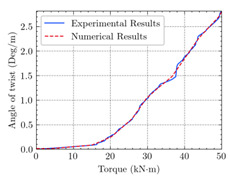 (b)	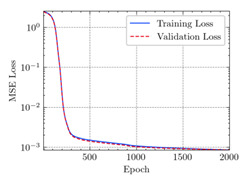 (c)	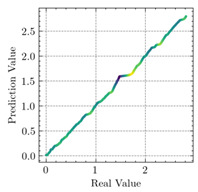 (d)
	Shear	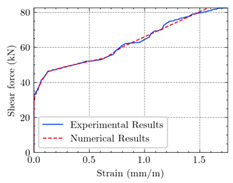 (e)	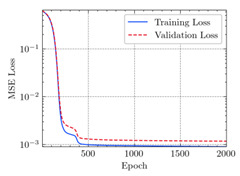 (f)	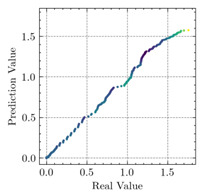 (g)
**Beam**	TB1S3	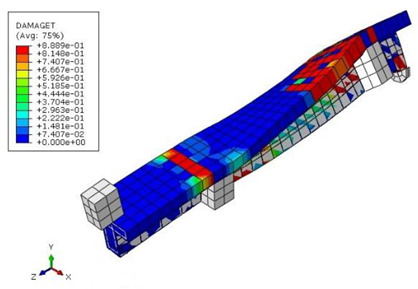 (a)		
**Structural Response**	Torsion	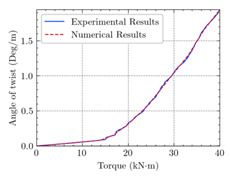 (b)	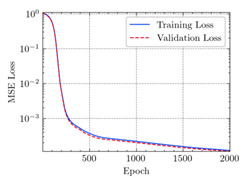 (c)	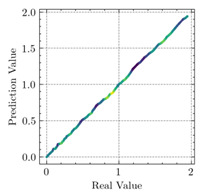 (d)
	Shear	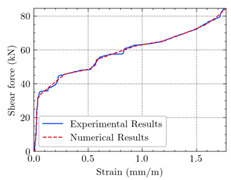 (e)	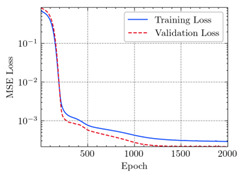 (f)	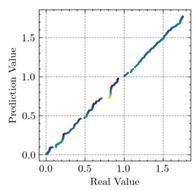 (g)
**Beam**	TB3	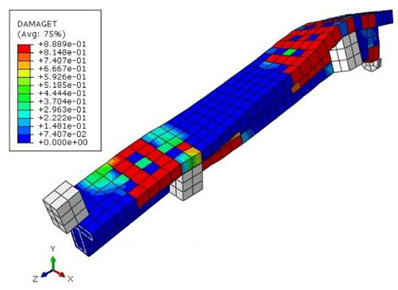 (a)		
**Structural Response**	Torsion	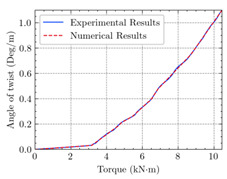 (b)	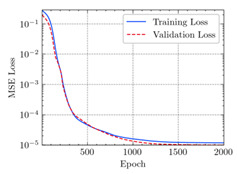 (c)	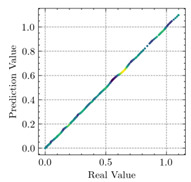 (d)
	Shear	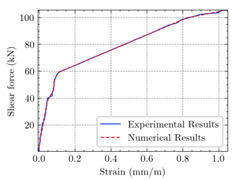 (e)	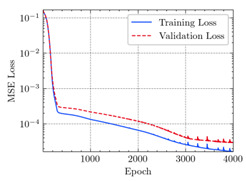 (f)	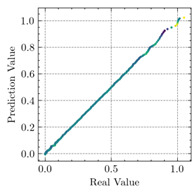 (g)
**Beam**	TB3S4	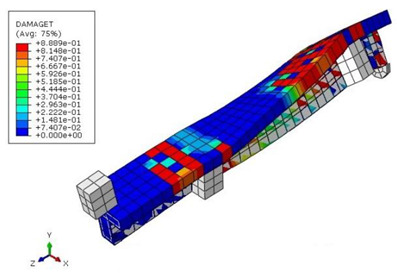 (a)		
**Structural Response**	Torsion	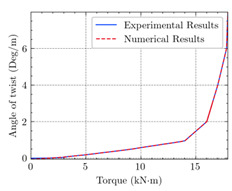 (b)	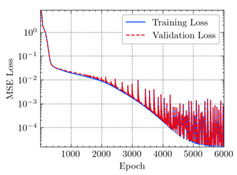 (c)	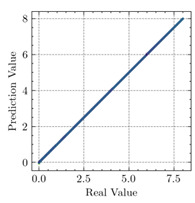 (d)
	Shear	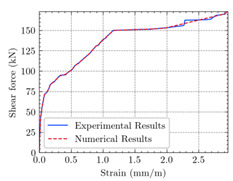 (e)	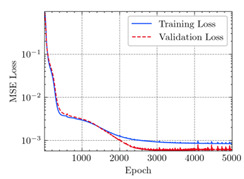 (f)	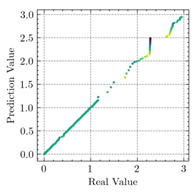 (g)

**Table 8:** Evaluation of the ANN model.

Structural Loading	Beam Model	Evaluation Index					
		Training Set		Test Set		All Set	
		*MSE*	*R* ^2^	*MSE*	*R* ^2^	*MSE*	*R* ^2^
Torsion	TB1	0.000218	0.999584	0.000208	0.999572	0.000216	0.999582
	TB1S1	0.000010	0.999942	0.000010	0.999934	0.000010	0.999941
	TB1S2	0.000855	0.998568	0.000853	0.998567	0.000854	0.998568
	TB1S3	0.000120	0.999586	0.000113	0.999617	0.000119	0.999592
	TB3	0.000012	0.999880	0.000010	0.999861	0.000011	0.999879
	TB3S4	0.000015	0.999997	0.000017	0.999996	0.000016	0.999997
Shear	TB1	0.000204	0.999619	0.000130	0.999742	0.000189	0.999643
	TB1S1	0.000282	0.998268	0.000196	0.998732	0.000265	0.998359
	TB1S2	0.000894	0.996909	0.001667	0.996277	0.000949	0.996775
	TB1S3	0.000291	0.999200	0.000214	0.999500	0.000276	0.999270
	TB3	0.000016	0.999808	0.000030	0.999677	0.000019	0.999780
	TB3S4	0.000846	0.998700	0.000617	0.998963	0.000800	0.998749

**Table 9:** Weights and biases of the developed ANN model.

Layers	Variables	Weights				Biases			
Input Layer– Hidden Layer 1	$w^{1}$ $\;\mathrm{and}\;b^{1}$	0.9055	0.1368	−2.1926	0.3227	0.1869	0.2014	0.2081	0.0113
		−0.4744	−0.5172	0.2766	0.4934	0.2912	0.2610	0.0833	−0.1754
Hidden Layer 1– Hidden Layer 2	$w^{2}$ $\;\mathrm{and}\;b^{2}$	−0.4782	−0.2169	−0.2380	−0.2013				
		−0.2345	0.5501	0.1987	0.8133				
		0.3647	0.1677	0.5850	0.5514				
		0.4934	−0.3571	0.1958	0.6721				
		0.4643	−0.9118	−0.1101	−0.2964				
		−0.5442	0.0397	−1.9594	0.9148				
		0.1914	0.4342	−0.0231	−0.3457				
		0.8302	−0.8049	0.1371	0.2899	0.0000	0.2942	0.0000	0.2942
		0.2652	−0.0362	−0.1578	−0.5761	0.1679	−0.0291	0.1679	−0.0291
		−0.0078	0.6746	0.3158	−1.9715				
		0.1724	0.0492	0.7593	−0.0956				
		0.1656	0.2187	1.2919	−0.0056				
		0.4801	0.4057	−0.6696	−0.1514				
		0.4514	0.8221	−0.4599	0.4107				
		0.4930	0.0647	0.5120	−1.7222				
		−0.1700	−0.4075	0.0203	0.4248				
Hidden Layer 2– Hidden Layer 3	$w^{3}$ $\;\mathrm{and}\;b^{3}$	−0.0330	0.8583	0.2370	−0.5885				
		0.2429	−0.4196	−0.8351	−0.1537				
		−0.2964	0.1166	−0.0714	−0.8275				
		0.0003	0.2155	−0.1605	0.2042				
		0.2977	−0.6194	0.6551	−0.1769				
		−0.1414	−0.5998	0.1234	0.1651				
		0.3675	0.0010	−0.0161	0.9348				
		0.1266	0.0796	−0.0199	−0.3759	0.218	0.2706	0.218	0.2706
		−0.4590	0.3589	−0.3686	0.2192	−0.2659	0.0000	−0.2659	0.0000
		0.8897	0.2254	−0.1640	0.0363				
		−1.4055	0.1166	0.6798	−0.2132				
		0.0544	0.1966	−0.1423	−0.8834				
		−0.9197	0.4698	−1.0743	0.2524				
		−0.3382	−0.3060	0.4118	−0.3654				
		0.3631	0.3432	0.4255	−0.0669				
		0.2511	−0.3471	0.4713	0.0675				
Hidden Layer 3– Hidden Layer 4	$w^{4}$ $\;\mathrm{and}\;b^{4}$	−0.0978	0.3359	−0.0234	−0.2039				
		0.3257	−0.5964	0.1834	0.1567				
		0.1030	0.0933	−0.3127	0.2791				
		0.6889	−0.1012	0.1629	0.0561				
		0.3922	0.3585	0.6967	0.0493				
		−0.0747	1.6836	−0.2026	0.5889				
		−0.1932	0.2583	0.3790	−0.098				
		−0.1582	0.2372	0.0852	0.5036	−0.0060	0.1727	−0.006	0.1727
		0.0680	0.1110	−0.3814	−0.0323	0.1675	−0.0533	0.1675	−0.0533
		0.0911	0.1246	−0.2549	−0.3707				
		−1.0282	−0.0705	0.5200	0.1239				
		0.4415	0.3857	0.0975	0.1955				
		0.1195	−0.0460	0.2357	0.2504				
		−0.2813	−0.4536	−0.3516	−0.4265				
		0.0556	0.0410	−0.4205	0.2033				
		−0.2370	−0.5362	−0.4228	−0.2052				
Hidden Layer 4– Output Layer	$w^{5}$ $\;\mathrm{and}\;b^{5}$	−1.1736	0.5198	−1.1736	0.5198	0.1462			
		0.6921	-0.6953	0.6921	−0.6953				
